# Dendritic Cell-induced Activation of Latent HIV-1 Provirus in Actively Proliferating Primary T Lymphocytes

**DOI:** 10.1371/journal.ppat.1003259

**Published:** 2013-03-21

**Authors:** Renée M. van der Sluis, Thijs van Montfort, Georgios Pollakis, Rogier W. Sanders, Dave Speijer, Ben Berkhout, Rienk E. Jeeninga

**Affiliations:** 1 Laboratory of Experimental Virology, Department of Medical Microbiology, Centre for Infection and Immunity Amsterdam (CINIMA), Academic Medical Centre, University of Amsterdam, Amsterdam, The Netherlands; 2 Department of Microbiology and Immunology, Weill Medical College of Cornell University, New York, New York, United States of America; 3 Department of Medical Biochemistry, Academic Medical Center, University of Amsterdam, Amsterdam, The Netherlands; Emory University, United States of America

## Abstract

HIV-1 latency remains a formidable barrier towards virus eradication as therapeutic attempts to purge these reservoirs are so far unsuccessful. The pool of transcriptionally silent proviruses is established early in infection and persists for a lifetime, even when viral loads are suppressed below detection levels using anti-retroviral therapy. Upon therapy interruption the reservoir can re-establish systemic infection. Different cellular reservoirs that harbor latent provirus have been described. In this study we demonstrate that HIV-1 can also establish a silent integration in actively proliferating primary T lymphocytes. Co-culturing of these proliferating T lymphocytes with dendritic cells (DCs) activated the provirus from latency. Activation did not involve DC-mediated C-type lectin DC-SIGN signaling or TCR-stimulation but was mediated by DC-secreted component(s) and cell-cell interaction between DC and T lymphocyte that could be inhibited by blocking ICAM-1 dependent adhesion. These results imply that circulating DCs could purge HIV-1 from latency and re-initiate virus replication. Moreover, our data show that viral latency can be established early after infection and supports the idea that actively proliferating T lymphocytes with an effector phenotype contribute to the latent viral reservoir. Unraveling this physiologically relevant purging mechanism could provide useful information for the development of new therapeutic strategies that aim at the eradication of HIV-1 reservoirs.

## Author Summary

Combination therapy can suppress the viral load in HIV-1 infected individuals to undetectable levels, but does not lead to complete virus eradication. Even after many years of successful therapy the virus is still present in long-lived cells as a latently integrated provirus. HIV-1 can re-establish systemic infection from this reservoir when therapy stops. Purging attempts in patients have been unsuccessful and HIV-1 latency remains a formidable barrier to virus eradication. Different cellular reservoirs that harbor latent HIV-1 proviruses have been described to consist mainly of resting memory T lymphocytes. Yet how this reservoir in memory T lymphocytes is established is still unclear as infection of these cells is very inefficient. In this paper we demonstrate that HIV-1 can establish a latent provirus in activated effector T lymphocytes. We observed that for every virus producing cell there is at least one other cell harboring a latent provirus, illustrating that latent infections occur frequently. Proliferating T lymphocytes are generally short-lived and their contribution to the total cellular reservoir thus seems limited. However, these activated T lymphocytes can revert into resting memory T lymphocytes and become part of the long-lived viral reservoir.

## Introduction

Combined antiretroviral therapy (cART) is able to suppress the HIV-1 plasma RNA load in patients to undetectable levels. The treatment, however, does not lead to complete virus eradication. Even after many years of successful cART the virus can rebound from latently infected cellular reservoirs and re-establish systemic infection upon therapy interruption [1]–[5]. Proviral latency is an effective strategy to sustain long-term infection by evading the immune system as long as viral antigens are not expressed and presented. Cells latently infected with HIV-1 remain a formidable barrier towards virus eradication and therapeutic attempts to purge these reservoirs have thus far been unsuccessful [6]–[8].

The pool of latent proviruses is established early during infection and forms a steady source of integrated proviral DNA lasting a lifetime for infected individuals [9], [10]. Early onset of cART reduces the size of the viral reservoir but does not prevent its formation [11]. HIV-1 establishes latent proviral integration mainly in T lymphocytes, but viral reservoirs in monocytes and dendritic cells have also been described [12]–[16].

How the reservoir in memory T lymphocytes is established remains unclear. Infection of quiescent memory T lymphocytes is inefficient due to incomplete reverse transcription and integration [17], [18]. Linear non-integrated cDNA is rapidly degraded with a half life of approximately 1 day, suggesting that *de novo* infection of memory T lymphocytes is unlikely to play a major role in formation of this long-lived viral reservoir [18]. However, it has been shown that cytokine stimulation of quiescent T lymphocytes can increase the HIV-1 infection efficiency by boosting reverse transcription and integration processes without inducing cell proliferation or up-regulation of cellular activation markers [19]–[24]. These integrated HIV-1 proviruses are transcriptionally insufficiently active to support the production of new viral particles and the resting T lymphocyte may thus become part of the long-lived latent reservoir. An alternative hypothesis for the formation of the latent reservoir is that actively proliferating T lymphocytes become infected with a transcriptionally silent provirus [25]–[27]. This latently infected proliferating T lymphocyte will not be recognized by the immune system and the proliferating cell can revert to a memory T lymphocyte, thus contributing to the long-lived viral reservoir.

We and others previously demonstrated that silent HIV-1 proviral integrations occur in T cell lines [28]–[35]. In this study, the presence of latent proviruses in primary proliferating T lymphocytes was studied. To show that silent integration does not equal a defective provirus, one should demonstrate that the provirus can be purged out of latency. Conventional anti-latency treatments, such as TNFα that is effective in T cell lines, had no effect on the latent provirus in actively proliferating primary T lymphocytes, in agreement with the results of other groups [36], [37]. Therefore, an alternative anti-latency treatment was explored. Co-culturing of the actively proliferating T lymphocytes with dendritic cells (DCs) was found to trigger a robust activation of the latent provirus.

Our results demonstrate that a natural mechanism based on cell-cell contact can purge HIV-1 from latency and support the idea that actively proliferating T lymphocytes contribute to the latent viral reservoir. Understanding the natural mechanisms that activate latent HIV-1 provirus may lead to novel intervention therapies to overcome latency.

## Results

### Latent HIV-1 in primary PHA-activated T lymphocytes can not be activated by classical anti-latency compounds

**Figure 1:**
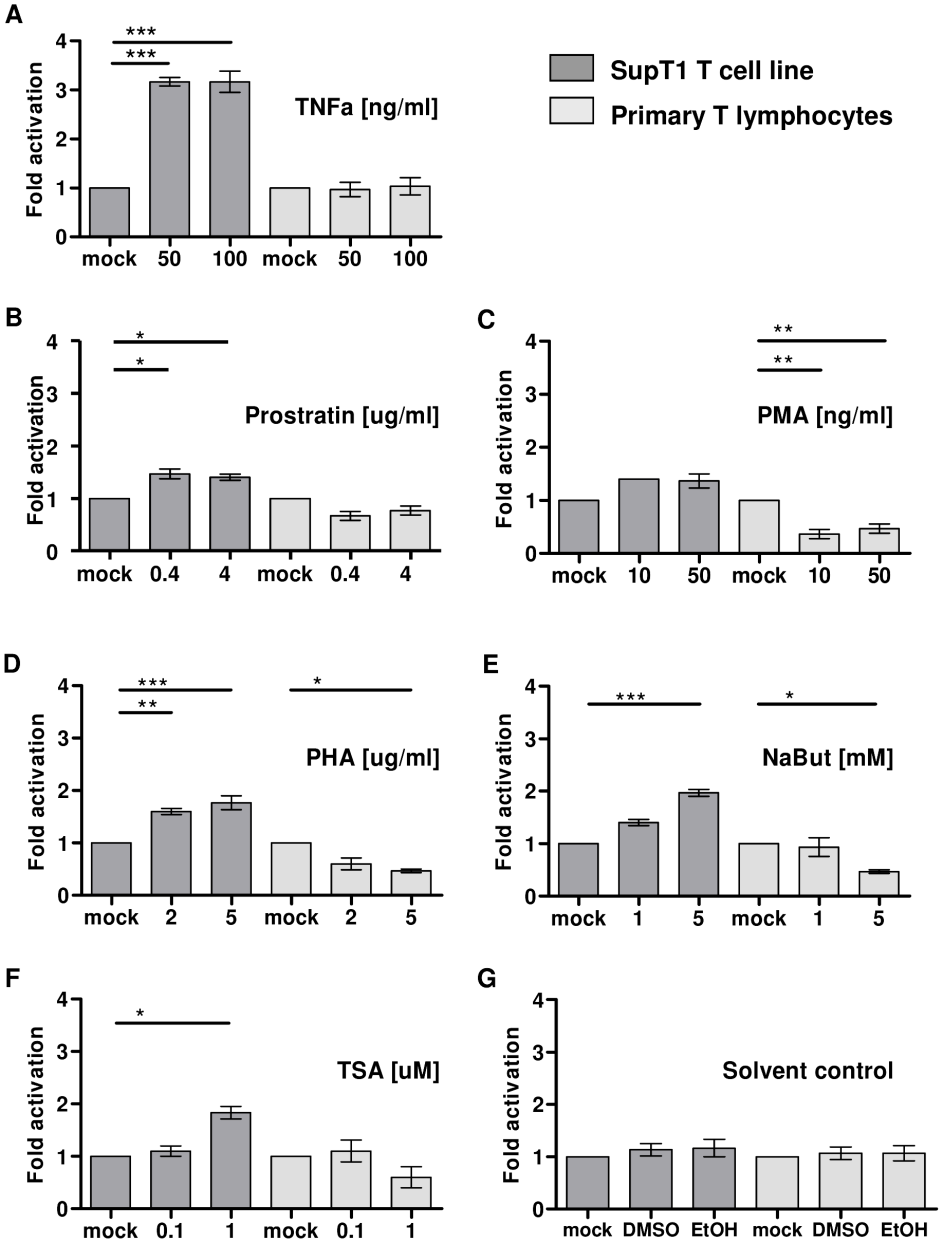
Latent HIV-1 in primary PHA-activated T lymphocytes can not be activated by classical anti-latency compounds. The SupT1 T cells (dark grey bars) and primary T lymphocytes (light grey bars) were infected with HIV-1 for 4 h. To prevent new rounds of virus replication, cells were cultured with the fusion inhibitor T1249. The HIV-1 infected cell culture was split 24 h after infection and maintained in the presence or absence of different latency activation drugs at the indicated concentrations for 24 h. The cells were stained and gated for CD3- and intracellular CA-p24-positivity with FACS flow cytometry. Activation of latent provirus is indicated as fold activation by comparing the treated cells with the control cells (**A** to **F**). Because NaBut and TSA are dissolved in DMSO and Prostratin in ethanol the cell cultures were also treated with either DMSO or ethanol (EtOH) as controls (**G**). The result shown is the mean fold activation (± standard error of the mean (sem)) of a representative experiment with a single donor performed in triplicate (n = 3).

To study HIV-1 proviral latency, the transcriptionally silent provirus must be distinguished from a defective provirus. This can be achieved by purging the silent provirus out of latency and measuring production of viral proteins such as the major structural protein Gag or its CA-p24 domain. We previously developed a latency assay and demonstrated that TNFα, which is a strong activator of the transcription factor NF-κB, could purge HIV-1 out of latency in the SupT1 T cell line [28], [35]. We reported that HIV-1 frequently establishes latent infection in these actively dividing T cells. Here we used this assay to test several known anti-latency drugs on *primary* PHA-activated T lymphocytes. As expected, treatment of HIV-1 infected SupT1 cells with TNFα yielded a 3-fold increase in the percentage of CA-p24 positive cells, but no such effect was observed in primary T lymphocytes (Fig. 1A). The phorbol esters prostratin and PMA can indirectly increase HIV-1 transcription via activation of the protein kinase C (PKC) signaling route [38], [39]. Treatment of infected SupT1 cells with prostratin increased the percentage of CA-p24 positive cells 1.5-fold, whereas no or even a small negative effect was observed for primary T lymphocytes (Fig. 1B). PMA treatment reduced the percentage of CA-p24 positive primary T lymphocytes while it did not change the percentage of CA-p24 positive SupT1 cells (Fig. 1C). Activation of the PKC route by stimulating the T cell receptor (TCR) with the cross-linker phytoheamagglutinin (PHA) increased the number of CA-p24 positive SupT1 cells 1.7-fold, but like PMA reduced the percentage of CA-p24 positive primary T lymphocytes (Fig. 1D).

Other activators of latent HIV-1 provirus in T cell lines include histone deacetylase (HDAC) inhibitors, such as sodium butyrate (NaBut) and trichostatin A (TSA) [40], [41]. These compounds prevent deacetylation of histone tails thereby creating a more open DNA chromatin conformation and this improves the accessibility of the HIV-1 promoter in the long terminal repeat (LTR) for transcription factors. In SupT1 cells, both NaBut and TSA activated latent provirus 2-fold when used at the highest concentration, but the compounds had no effect on the percentage of CA-p24 positive primary T lymphocytes (Fig. 1D and E). DMSO and ethanol, used to dissolve NaBut and TSA, did not affect transcriptional activity of latent provirus (Fig. 1F). These results indicate that many of the known anti-latency drugs can indeed purge HIV-1 out of latency in the SupT1 T cell line but *not* in primary proliferating T lymphocytes. Thus, either PHA-activated primary T lymphocytes do not harbor latent HIV-1 infections, or the anti-latency drugs used at the indicated concentrations are not sufficient to activate HIV-1 provirus from latency in these primary cells.

### Latent HIV-1 provirus in primary PHA-activated T lymphocytes can be activated by contact with dendritic cells

**Figure 2:**
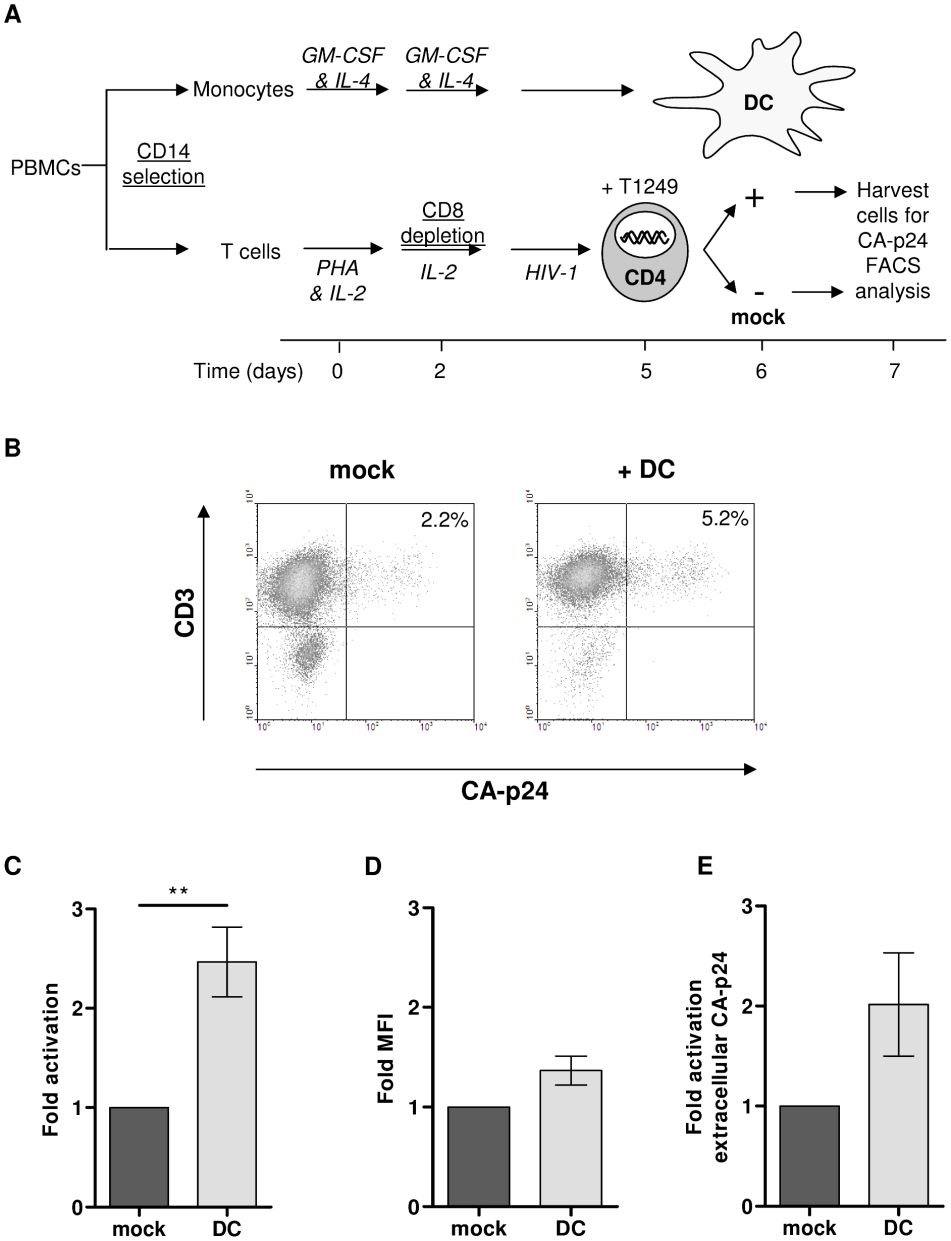
Latent HIV-1 provirus in primary PHA-activated T lymphocytes can be activated by dendritic cells. **A**: Schematic time line of the latency assay. PBMCs were isolated from a healthy blood donor. The monocytes and T lymphocytes were separated by CD14 selection. Monocytes were differentiated into monocyte-derived dendritic cells (DC) and CD4-enriched T lymphocytes were infected with HIV-1. 4 h post infection excess virus was washed away and the fusion inhibitor T1249 was added to prevent spreading of infection. The infected T lymphocytes were either co-cultured with DCs or not and the cells were analyzed with FACS flow cytometry for CD3- and intracellular CA-p24-positivity. **B**: Representative dot-plot of T lymphocytes positive for CA-p24 and CD3 with or without DC co-culturing. **C**: Analyses of the percentage of CA-p24 positive T lymphocytes shown as the mean fold activation. **D**: Analyses of the CA-p24 MFI shown as the mean fold activation. **E**: Virus particle secretion from infected T lymphocytes was determined by measuring CA-p24 in the culture supernatant with ELISA and the fold activation was calculated. All results presented are the mean values (± sem) of two independent experiments using two donors and each experiment was performed in triplicate (n = 6).

Dendritic cells (DCs) regulate T and B cell responses via cell-cell contact in combination with secretion of specific cytokines and chemokines [42], [43]. To investigate if a more physiological cell-based stimulus could activate HIV-1 from latency in primary T lymphocytes, the cells were co-cultured with immature monocyte-derived dendritic (DCs). The HIV-1 infected T lymphocyte culture was split 24 h after infection into a mock treated culture and a co-culture with DC (Fig. 2A). In the latency assay new rounds of virus replication and virus transmission from DC to T lymphocyte are prevented by the fusion inhibitor T1249. The cells were harvested after 24 hours, stained for intracellular CA-p24 and analyzed by flow cytometry. The percentage of CA-p24 positive T lymphocytes increased significantly from 2.2% in the control culture to 5.2% upon co-culture with DCs (Fig. 2B). This 2.4-fold activation shows that HIV-1 can frequently establish a latent provirus early after infection of PHA-activated T lymphocytes and DCs can induce proviral gene expression from the silent provirus to re-initiate virus production (Fig. 2C). Similar results were obtained with CD3/CD28-activated T lymphocytes instead of PHA-activated T cells (Fig. S1).

To confirm that the PHA-activated T lymphocytes have an effector phenotype they were stained with different antibodies to detect immune phenotype markers by flow cytometry. The PHA-activated T lymphocytes expressed low levels of CD69, CD127 and CCR7 and high levels of CD25 and CD45RO, as expected of effector T lymphocytes (Fig. S2A). For most markers the expression level, measured with the mean fluorescent intensity (MFI), did not change as a result of virus production, except for a significant increase in CD25 and CD45RO expression in the CA-p24 positive T lymphocytes compared to CA-p24 negative cells (Fig. S2B). The increase in CD25 expression was even more pronounced in the CA-p24 positive T lymphocytes that were co-cultured with DCs (Fig. S2C and S2D).

To investigate whether DCs induce apoptosis of the HIV-1 infected T lymphocytes, the cells were analyzed for the presence of the phospholipid phosphatidylserine (PS), an early apoptosis marker. Co-culturing of T lymphocytes with DCs slightly increased the percentage of PS positive cells but this was observed for the complete T lymphocyte population and not specifically for the CA-p24 positive cells, demonstrating that increased CA-p24 expression is not caused by the onset of apoptosis (Fig. S3).

To investigate whether contact with DCs also enhances the virus production per cell, we inspected the MFI of CA-p24 positive T lymphocytes. The MFI of DC versus mock treated cells was compared and the ratio of the two values was calculated (Fig. 2D). There was no significant difference, indicating that virus production per individual T lymphocyte does not increase. To investigate the total virus production in the cell culture, secreted CA-p24 was measured in the culture supernatant with ELISA. Co-culturing of the T lymphocytes with DCs increased the extracellular CA-p24 production in the culture supernatant 2-fold, but this was not significantly different from the extracellular CA-p24 measured in the mock treated culture (Fig. 2E). When the extracellular CA-p24 production was corrected for the increased number of intracellular CA-p24 positive cells, no nett difference was observed (data not shown). These results demonstrate that DCs induce more T lymphocytes to produce HIV-1 but that virus production per cell does not change.

### DCs do not significantly activate latent HIV-1 provirus in the SupT1 T cell line

**Figure 3:**
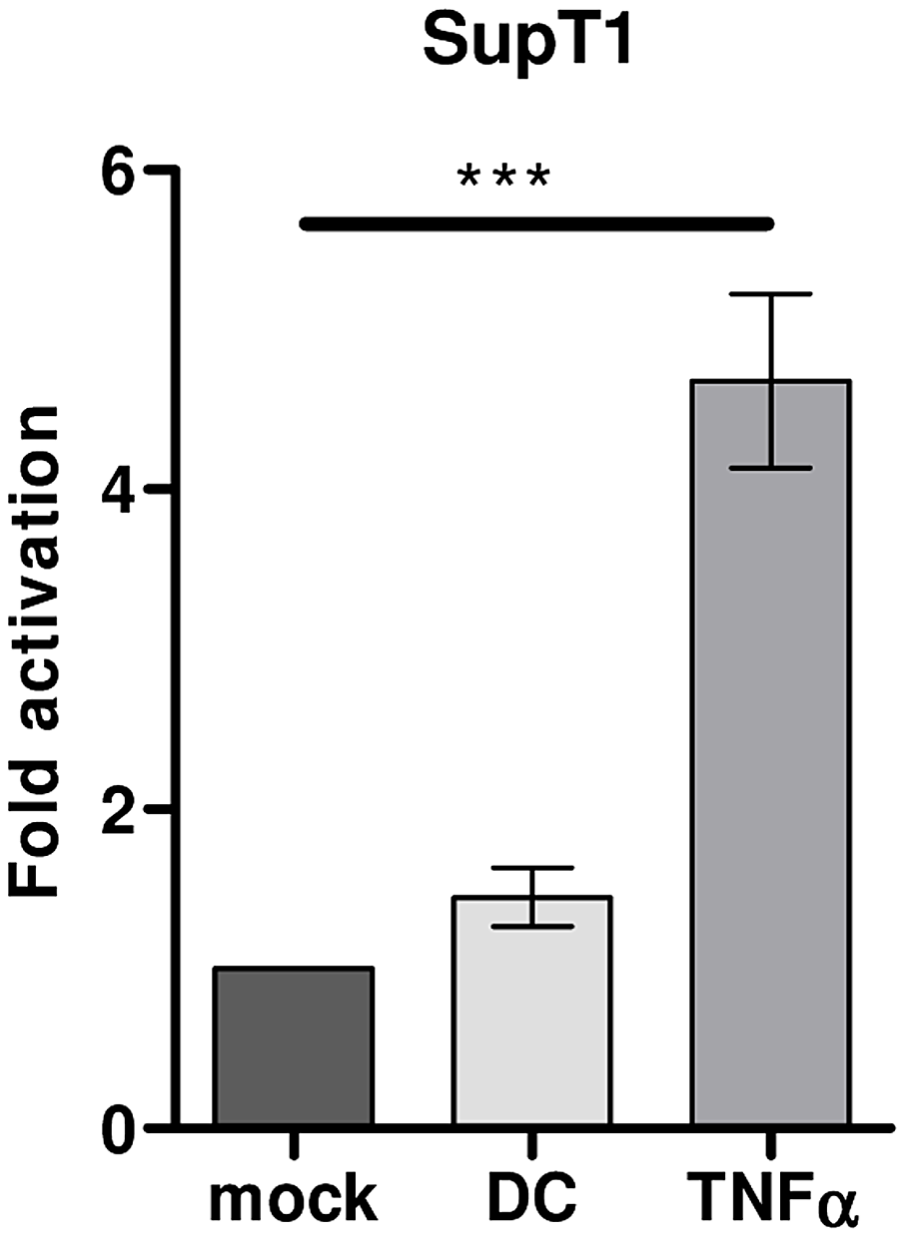
DCs do not activate latent HIV-1 provirus in SupT1 cells. HIV-1 inoculated SupT1 T cells were mock treated, treated with TNFα (50 ng/ml) or co-cultured with DCs (ratio DC∶SupT1 cell 1∶3) in the latency assay. The results presented are the mean values (± sem) that were obtained from two independent experiments with two donors and each experiment was done in triplicate (n = 6).

The conventional anti-latency drugs that can activate provirus from latency in SupT1 cells are insufficient to activate latent provirus in primary T lymphocytes. However, latent provirus in T lymphocytes can be activated upon co-culture with DCs. To investigate whether DCs can also activate HIV-1 from latency in the SupT1 T cell line, HIV-1 infected SupT1 cells were mock treated or co-cultured with DCs for 24 hours. The percentage of CA-p24 positive SupT1 cells increased from an average of 2.7% in the mock treated culture to 4.1% in the co-culture with DCs. This 1.5-fold increase is not significantly different, as underscored by the comparison to the 4.7-fold activation upon TNFα treatment (Fig. 3). These combined results indicate that distinct pathways seem to trigger activation of latent provirus in SupT1 T cells versus primary T lymphocytes.

### HIV-1 latency in activated T lymphocytes is maintained over time

**Figure 4:**
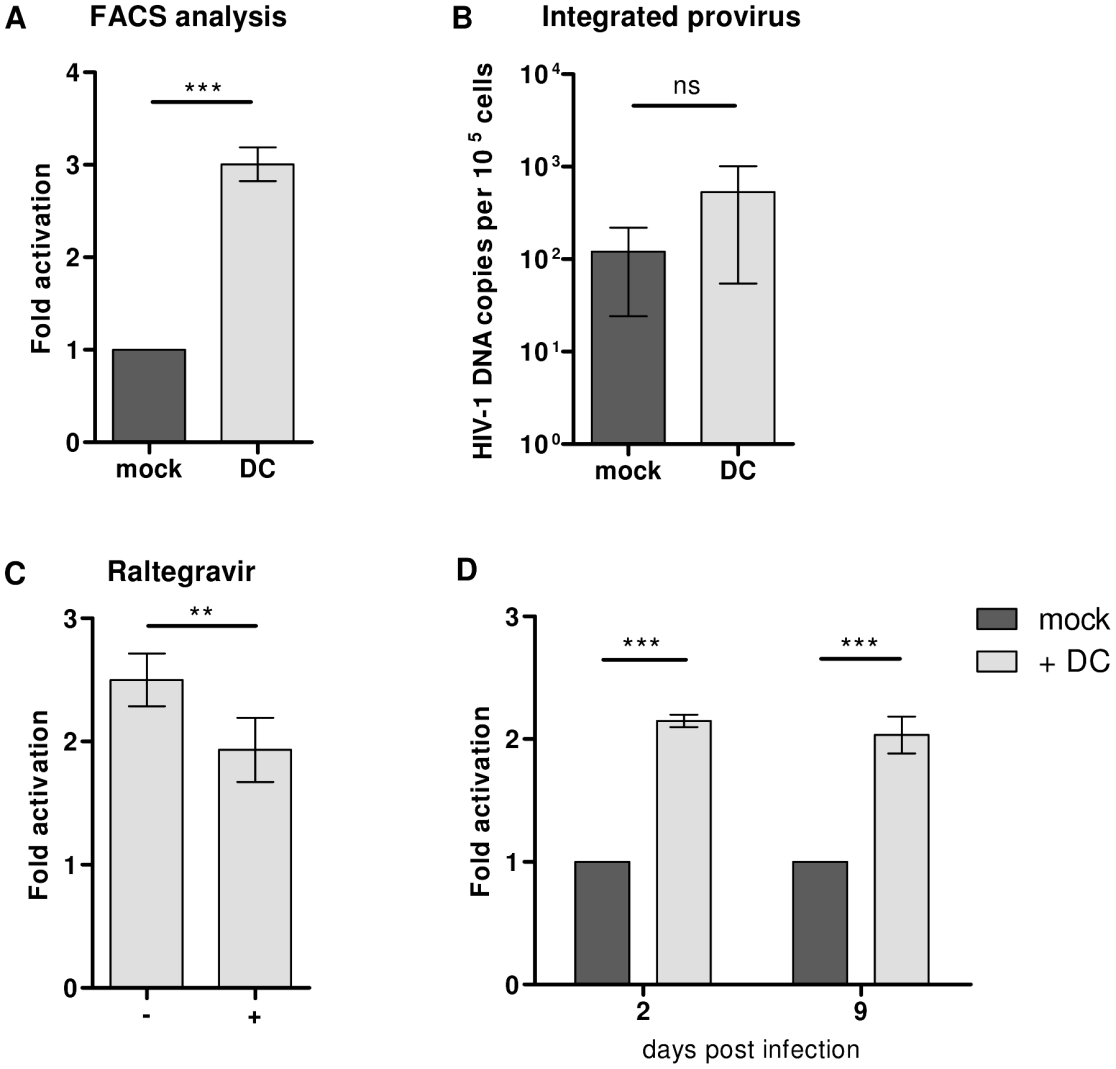
HIV-1 latency in activated T lymphocytes is maintained over time. **A**: An aliquot of infected T lymphocyte cultured with or without DCs was analyzed for intracellular CA-p24 expression by FACS flow cytometry and the fold activation was calculated. The remainder of the cell culture was collected and analyzed by TaqMan. **B**: Quantitative TaqMan assay to measure total amount of integrated provirus (see text). The cellular β-actin house keeping gene was measured as input control. The results presented are the mean values (± sem) that were obtained from three independent experiments with three different donors and each experiment was performed in triplicate (n = 9) **C**: T lymphocytes were infected for 4 hours, washed to remove excess virus, and cultured with the fusion inhibitor T1249, as usual, or cultured with T1249 combined with the integration inhibitor Raltegravir. 24 hours post infection both cultures were split into a mock treated culture or co-culture with DCs. After another 24 hours cells were harvested, analyzed for intracellular CA-p24 expression by FACS flow cytometry, and fold activation was calculated. The results presented are the mean values (± sem) that were obtained from two independent experiments with two different donors and each experiment had 3 replicates (n = 6) **D**: CD3/CD28-activated T lymphocytes were infected according to the standard latency assay, except that the culture was split into 3 cultures on day 2 post infection. The first culture was mock treated, the second co-cultured with DC's and the third was maintained for 1 week. The mock treated and DC co-cultured T lymphocytes were harvested after 24 hours and analyzed for intracellular CA-p24 expression by flow cytometry. On day 9 post infection the third culture was split into two cultures; one mock treated and one co-cultured with DC's. Both were harvested after 24 hours and analyzed by flow cytometry to calculate the fold activation. The results presented are the mean values (± sem) that were obtained from two independent experiments using two different donors and each experiment was done in triplicate (n = 6).

In the latency assay DCs are added to T lymphocytes 24 hours after a single round HIV-1 infection to allow for completion of the reverse transcription and integration processes. Several control experiments were performed to investigate if DCs influence delayed reverse transcription or integration processes rather than transcriptional activation of latent HIV-1 provirus. First, the integrated DNA copy numbers were analyzed. Infected T lymphocytes were co-cultured with DCs or mock treated and an aliquot of the cultures was analyzed for CA-p24 expression by flow cytometry, which showed the expected 3-fold induction as the percentage of CA-p24 positive cells increased from 2.8% in the mock treated culture to 8.6% in the DC co-culture (Fig. 4A). The remaining cells were pelleted, subjected to proteinase K treatment, and the HIV-1 DNA copy number was analyzed with a TaqMan assay that detects the number of integrated HIV-1 copies with primers binding to repetitive chromosomal *Alu* segments in combination with primers specific for HIV-1 DNA. The T lymphocytes co-cultured with DCs appeared to have higher integrated viral copy numbers compared to the mock treated culture, but this trend was not statistically significant (Fig. 4B). To further monitor the effects on integration, the latency assay was done in the presence or absence of the integrase inhibitor Raltegravir, which was added at the start of the DC co-culture. Raltegravir caused a small but significantly reduction of the DC-mediated provirus activation. Nevertheless, the 2-fold induction of latent provirus remained, showing that DCs can influence the HIV-1 integration process but also activate latent provirus (Fig. 4C). To eliminate the effect on the early events of the HIV-1 replication cycle, the latency assay was repeated 9 days after infection. At this time all reverse transcription and integration processes should be completed. To allow for prolonged culturing, the T lymphocytes were activated with beads coated with CD3 and CD28 antibodies instead of PHA and infected according to the standard latency assay, except that the culture was split into 3 cultures on day 2 post infection. The first culture was mock treated, the second co-cultured with DC's and the third was maintained for 1 week. The mock treated and DC co-cultured T lymphocytes were harvested after 24 hours and analyzed by flow cytometry. On day 9 post infection the third culture was split into two cultures; one mock treated and one co-cultured with DC's. Both were harvested after 24 hours and analyzed by flow cytometry. The percentage CA-p24 positive T lymphocytes increased 2-fold by DC co-culture executed on either day 2 or day 9 post infection (Fig. 4D). These results show that PHA-activated T lymphocytes still harbor latent provirus at one week after infection and that this provirus can become transcriptionally active upon T lymphocyte contact with DCs.

### PHA-activated T lymphocytes harbor latent HIV-1 provirus

**Figure 5:**
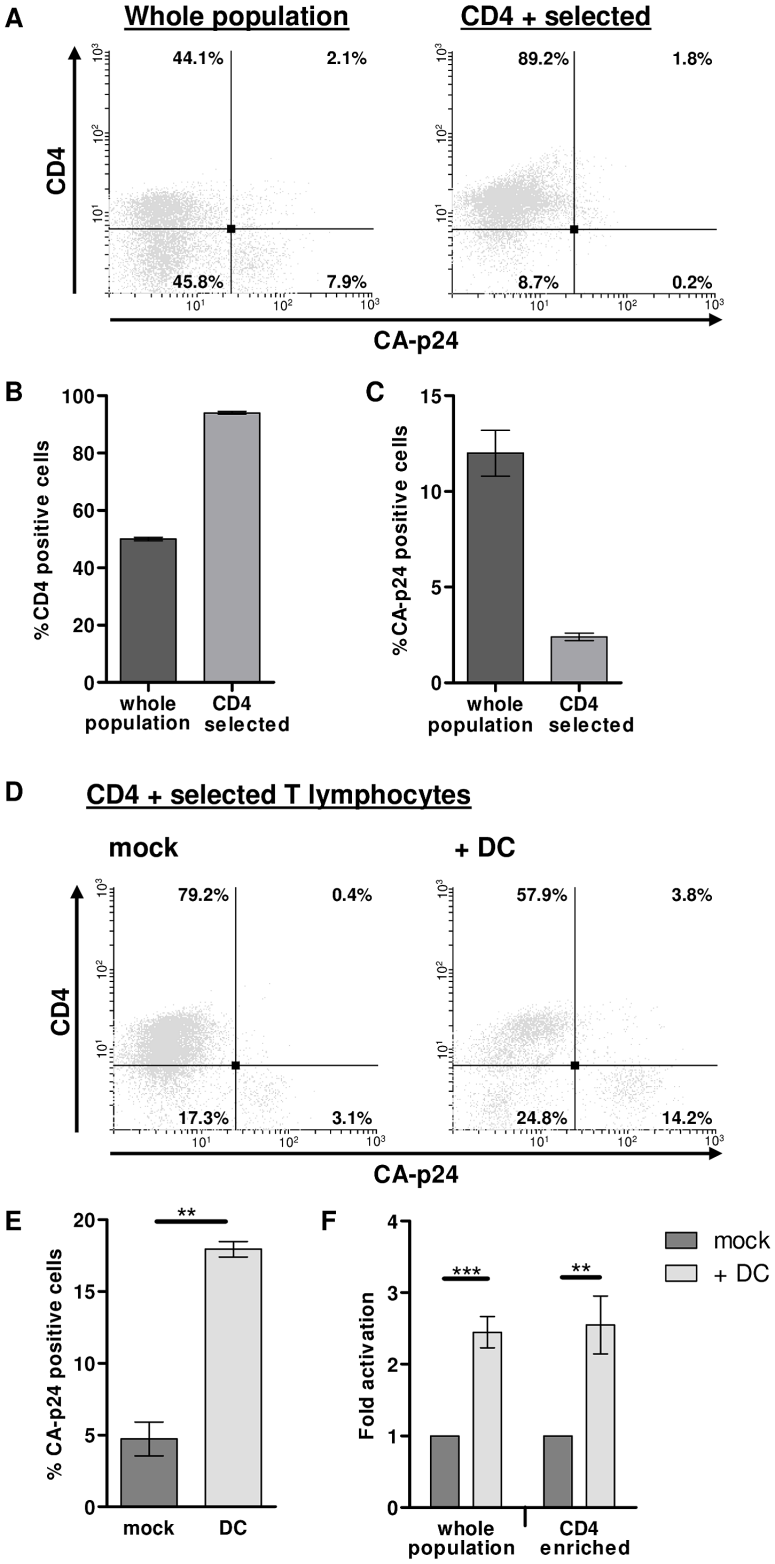
PHA-activated T lymphocytes harbor latent HIV-1 provirus. T lymphocytes harboring latent provirus maintain CD4 expression as the viral Nef protein downregulates CD4 expression in productively infected cells. PHA-activated T lymphocytes were infected according to the latency assay and selected for CD4 expression prior to co-culture with DCs or mock treatment. **A**: Representative dot-plot showing the CD4 and CA-p24 positive cells before and after CD4 selection. **B**: Percentage of CD4 positive T lymphocytes before and after CD4 selection. **C**: Percentage of CA-p24 positive T lymphocytes before and after CD4 selection. **D**: Representative dot-plot showing the CD4 and CA-p24 positive cell in the CD4 selected culture that was either mock treated or co-cultured with DCs. **E**: Percentage of CA-p24 positive T lymphocytes after 24 h mock treatment or co-culturing with DCs. Shown is a representative experiment with a single donor performed in duplicate (n = 2). **F**: Analyses of the percentage of CA-p24 positive T lymphocytes that were either mock treated or co-cultured with DCs, depicted as the mean fold activation. Results are obtained from 3 independent experiments with four different donors and each experiment was performed in duplicate (n = 8).

To unequivocally show that PHA-activated T lymphocytes harbor latent provirus the latency assay was performed with HIV-1 infected CD4 expressing T lymphocytes. Productively infected T lymphocytes down-regulate CD4 expression at the cell surface via the viral Nef protein, one of the early HIV-1 proteins, whilst latently infected T lymphocytes retain normal CD4 expression levels [44]. To study if DCs induce gene expression of truly latent provirus, PHA-activated T lymphocytes (mixed population) were infected with HIV-1 according to the latency assay. A single day after infection half of the T lymphocyte culture was used to select CD4 expressing cells with magnetic beads (CD4 selected, Fig. 5A). To determine selection efficiency, the percentage of CD4 expressing cells was compared by flow cytometry analysis. In a representative experiment starting with a cell population of which 50% expressed CD4, we could enrich up to 94% (Fig. 5B). As expected, the CD4 selected cells exhibited decreased CA-p24 positivity (Fig. 5C). Both mixed and CD4 selected T lymphocytes were co-cultured with DCs or mock treated for 24 hours, and the CD3 positive T lymphocyte population was analyzed for CD4 and CA-p24 expression by flow cytometry (Fig. 5D). Co-culturing the infected CD4 selected T lymphocytes with DCs induced the percentage of CA-p24 positivity to increase while the cells that became CA-p24 positive lost CD4 expression (Fig. 5E and data not shown). The average 2.6-fold DC-mediated activation of latent provirus in the CD4 selected T lymphocytes was similar to the 2.5-fold activation in the whole population T lymphocyte population (Fig. 5F). Thus, the latent HIV-1 provirus in PHA-activated T lymphocytes – assayed by unaffected CD4 expression – is sensitive to transcriptional activation upon T lymphocyte DC contact.

### DCs activate latent HIV-1 independent of donor variation

**Figure 6:**
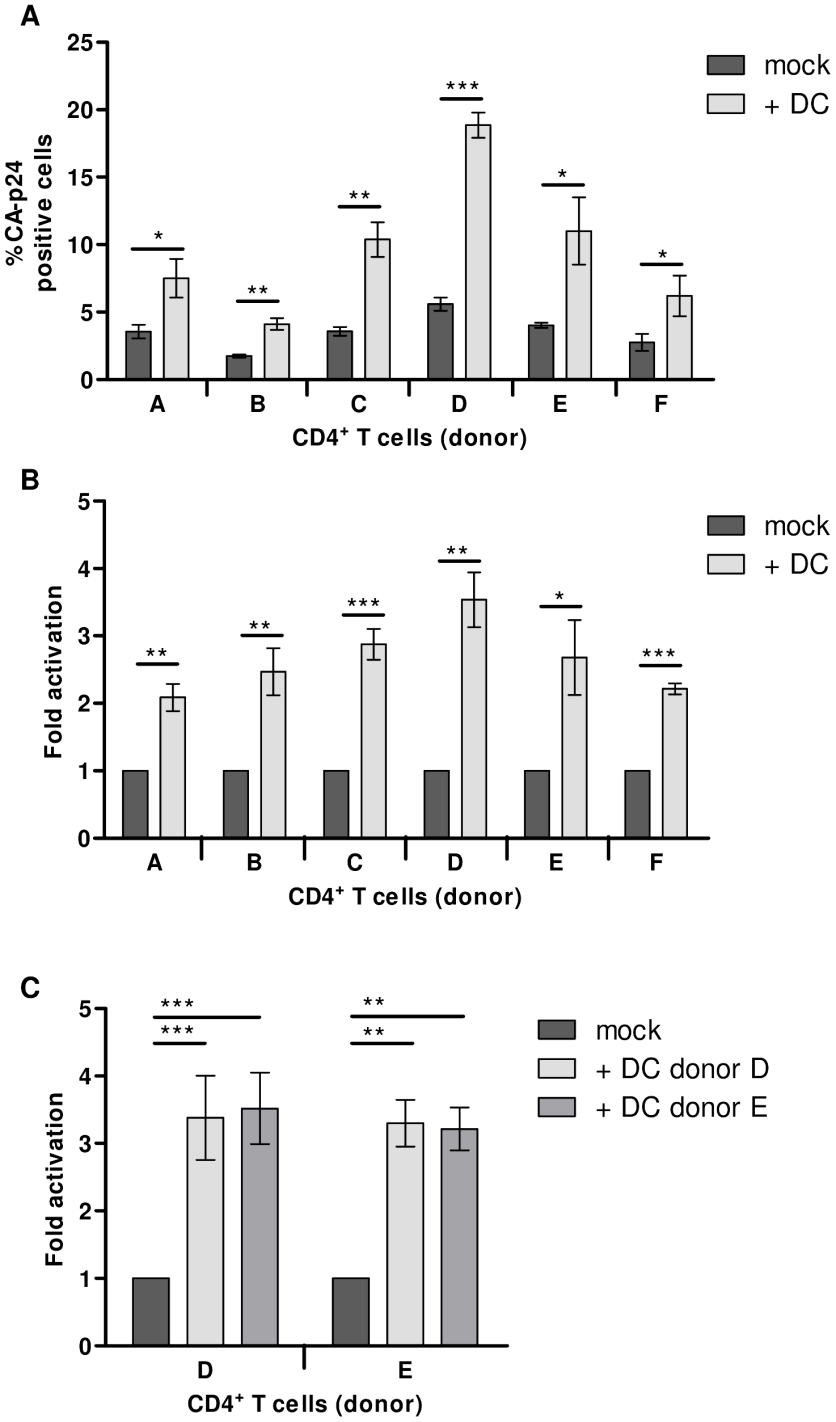
DCs activate latent HIV-1 independent of donor variation. **A**: CD4^+^ T lymphocytes from six healthy donors were examined in the HIV-1 latency assay. The percentage of intracellular CA-p24 positive T lymphocytes with or without DC co-culture was determined. **B**: HIV-1 fold latency activation in T lymphocytes from 6 different donors. The results are presented as the mean values (± sem) of three independent experiments each performed in triplicate (for each donor n = 6). **C**: HIV-1 inoculated CD4^+^ T cells either mock treated or co-cultured with either autologous or allogenic DCs. Results presented are mean values (± sem) obtained from two independent experiments performed in triplicate (n = 6).

To investigate possible differences in establishment and activation of latent proviruses between individuals, DCs and T lymphocytes were isolated from six healthy blood donors and tested in the latency assay. The percentage of mock treated CA-p24 positive T lymphocytes ranged from 1.7% for donor B to 5.6% for donor D (Fig. 6A). Despite such differences in infection rate, latent provirus activation by stimulation with autologous DCs was apparent for all donors. The DC-mediated fold activation from latency ranged from 2-fold for donor A to 3.5-fold for donor D (Fig. 6B). Next, we investigated whether latent HIV-1 proviruses in T lymphocytes could be activated by co-culturing with allogenic DCs. Infected T lymphocytes from donor D and E were mock treated or co-cultured with DCs from donor D or E. Both autologous and allogenic DCs similarly increased the percentage of CA-p24 positive T lymphocytes by 3-fold (Fig. 6C).

### DC-mediated HIV-1 activation is not induced via TCR stimulation

**Figure 7:**
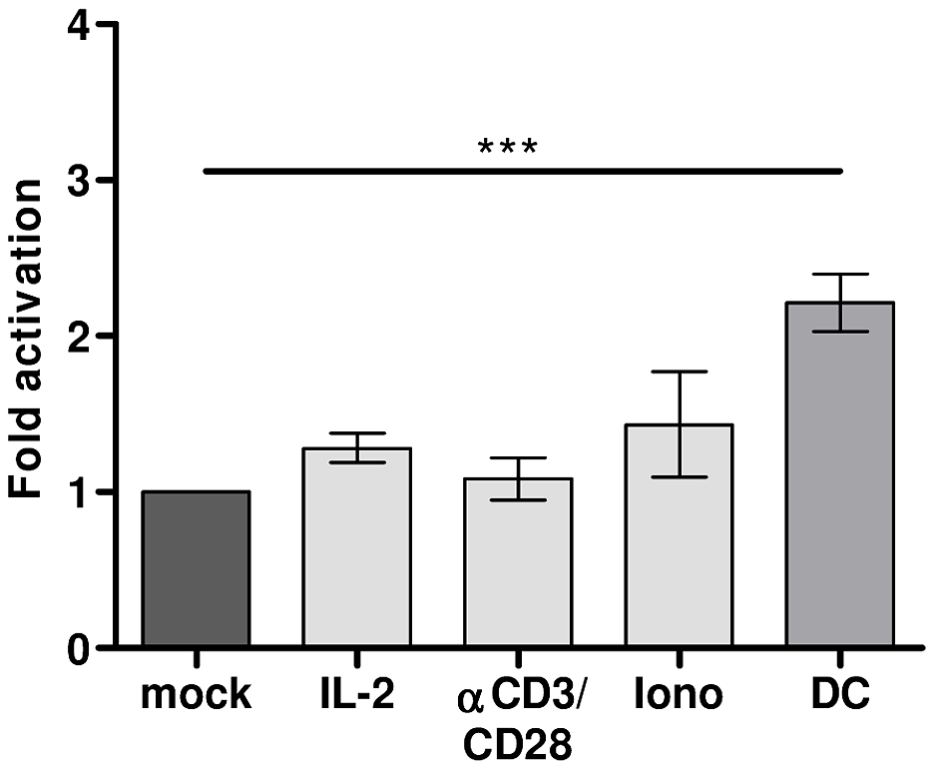
DC-mediated HIV-1 activation is not induced via TCR stimulation. HIV-1 infected T lymphocytes were mock treated, cultured with IL-2, αCD3/CD28 beads, ionomycin (iono) or co-cultured with DCs in the latency assay. The results presented are mean values (± sem) obtained from two independent experiments with two different donors, each experiment was performed in triplicate (n = 6).

It has previously been shown that activation of latent HIV-1 in quiescent memory T lymphocytes can be achieved by activating the cells via TCR stimulation with CD3/CD28 specific antibodies or using IL-2 or ionomycin [45]–[47]. Since the T lymphocytes in our latency assay are activated via TCR stimulation with PHA prior to HIV-1 infection, we tested whether further TCR stimulation could increase the number of CA-p24 positive cells. Reactivation with CD3/CD28 antibodies did not increase the percentage of CA-p24 positive cells (Fig. 7). Treating the T lymphocytes with IL-2 or ionomycin, which activates the NFAT transcription factor, also had no significant effect on the percentage of CA-p24 producing cells. The T lymphocytes used in this study are all TCR stimulated and still harbor latent provirus that can be activated by DCs. This demonstrates that TCR stimulation is not sufficient to activate all latent provirus in proliferating T lymphocytes.

### DC-SIGN does not play a role in DC-mediated HIV-1 activation from latency

**Figure 8:**
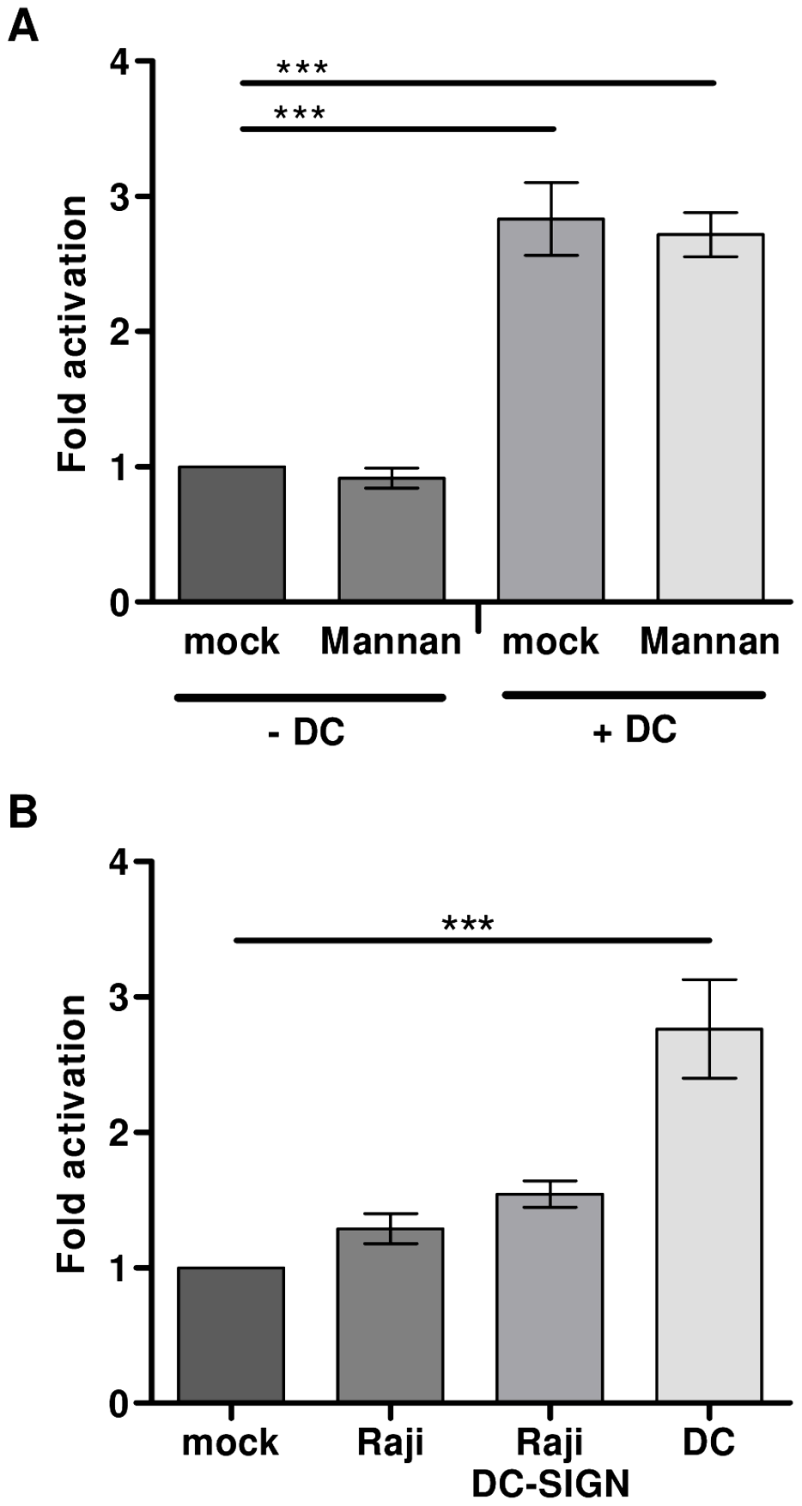
DC-SIGN does not play a role in DC-mediated HIV-1 activation from latency. **A**: HIV-1 infected T lymphocytes were mock treated or co-cultured with DCs in the presence or absence of the C-type lectin binding competitor mannan. Results presented are mean values (± sem) obtained from three independent experiments with two different donors (n = 9). **B**: HIV-1 infected T lymphocytes were co-cultured in the absence or presence of DCs, Raji, or Raji cells constitutively expressing DC-SIGN. Results presented are mean values (± sem) obtained with two independent experiments with 2 different donors (n = 6).

The C-type lectin DC-SIGN is a cell surface molecule expressed on DCs that facilitates HIV-1 infection of T lymphocytes *in cis* or *in trans* [48], [49]. Furthermore, DC-SIGN can induce HIV-1 transcription in DCs themselves [50]. To investigate whether DC-SIGN is also involved in the activation of latent HIV-1 provirus in primary T cells, the HIV-1 infected T lymphocytes were mock treated or co-cultured with DCs in the presence or absence of the C-type lectin competitor mannan (Fig. 8A). Addition of mannan did not influence DC-mediated provirus activation. To further study a possible role for DC-SIGN, T lymphocytes were co-cultured with either Raji or modified Raji cells expressing DC-SIGN (Raji-DC-SIGN). DCs triggered a 3-fold activation, whereas both the Raji and Raji-DC-SIGN cells could not purge provirus out of latency (Fig. 8B). The combined results indicate that DC-SIGN is most likely not involved in DC-mediated HIV-1 activation from proviral latency.

### DC-secreted factors and cell-cell contact contribute to activation of latent HIV-1

**Figure 9:**
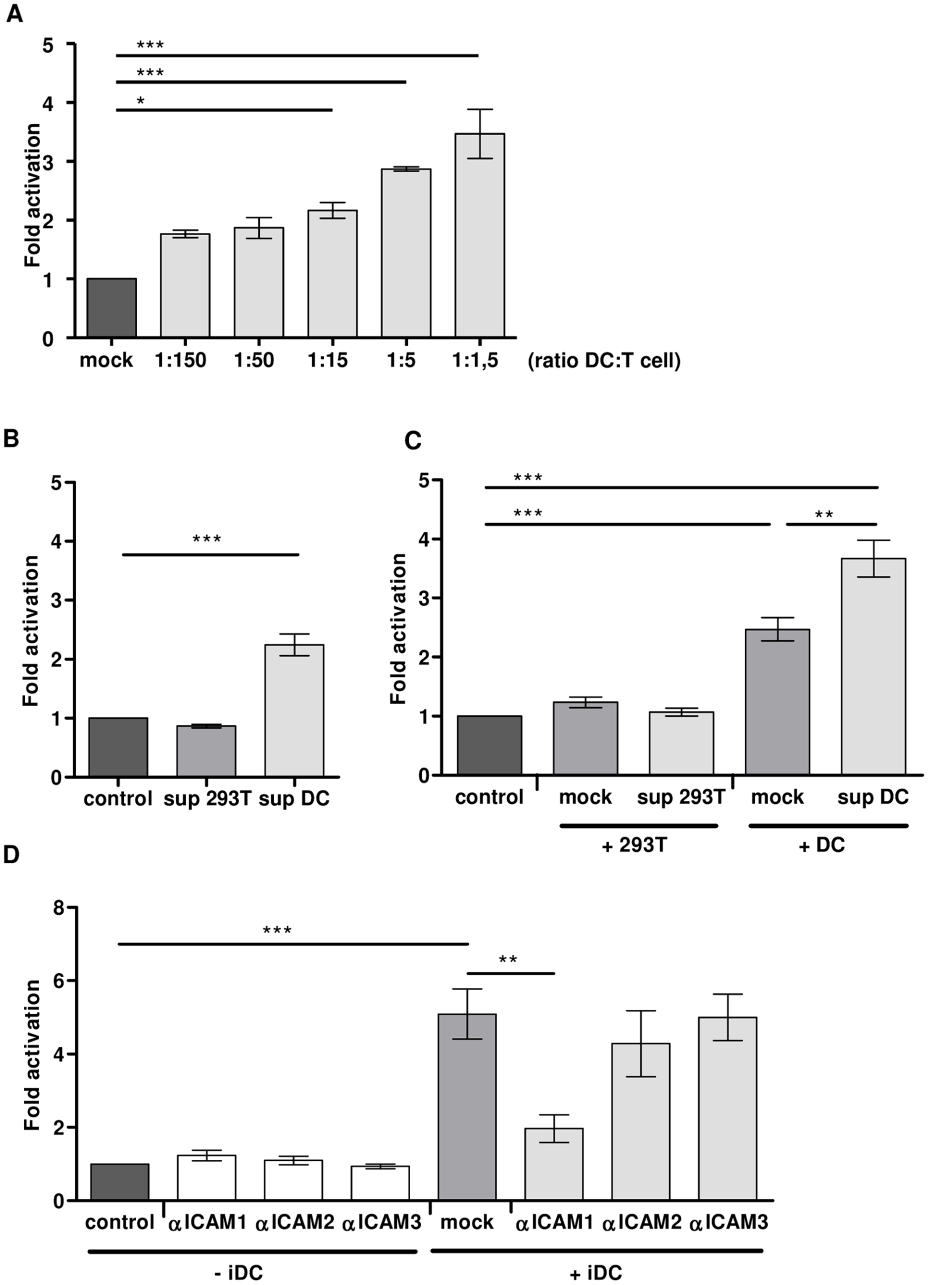
DC-secreted factors and cell-cell contact contribute to activation of latent HIV-1. **A**: HIV-1 infected T lymphocytes (150×10^3^) were mock treated or co-cultured with increasing numbers of DCs (1×10^3^–100×10^3^) in the latency assay. The results are mean values (± sem) obtained from a representative experiment performed in triplicate (n = 3). **B**: The HIV-1 infected T lymphocytes were inoculated with the cell-free supernatants of control HEK 293T cells, DCs, or fresh media (mock). Results are mean values (± sem) of three independent experiments with 2 different blood donors, each experiment was performed in triplicate (n = 9). **C**: HIV-1 infected T lymphocytes were co-cultured in the absence (‘control’) or presence of HEK 293T cells, or DCs resuspended in either fresh medium (‘mock’) or cultured supernatant. Results are mean values (± sem) of three independent experiments with 2 different blood donors, each experiment was performed in triplicate (n = 9). **D**: DC-mediated HIV-1 activation from latency in T lymphocytes in the presence or absence of ICAM specific antibodies. The results presented are mean values (± sem) of two independent experiments with 2 different blood donors, each experiment was performed in triplicate (n = 6).

To investigate the requirements for DC-mediated activation of latent provirus, we first studied the effect of co-culturing the T lymphocytes with increasing numbers of DCs (ratio DC∶T; 1∶150–1∶1,5). The CA-p24 positive T lymphocytes increased from an average of 1.4% in the mock treated culture to an average of 2.5% in the DC co-culture at a ratio of 1∶150, yielding a 1.8-fold activation of latent provirus. Increasing DC numbers enhanced proviral activation to 4.8% CA-p24 positive T lymphocytes, representing 3.5–fold activation with 1 DC per 1.5 T lymphocytes (Fig. 9A). To investigate the role of DC-secreted components, cell-free DC culture supernatant was added to infected T lymphocytes. As a control, cell-free culture supernatant of HEK 293T cells was used. The DC-supernatant induced a 2.2-fold activation whereas HEK 293T supernatant showed no activation (Fig. 9B). This result demonstrates that the DC culture medium contains (a) DC-secreted factor(s) activating latent HIV-1 provirus. This is not mediated by IL-4 or GM-CSF that are used to differentiate the monocytes into DCs as both cytokines did not induce activation of the latent provirus (Fig. S4). Co-culturing of infected T lymphocytes with freshly washed DCs, thus removing soluble factors, induced a 2.5-fold increase in percentage of CA-p24 positive cells, while washed DCs combined with DC supernatant gave the strongest (3.7-fold) activation (Fig. 9C). This demonstrates that a combination of DCs and their secreted factors induce the strongest activation. Next, we investigated if the activation of latent provirus can be inhibited by blocking the DC-T lymphocyte cell-cell interaction. Since TCR-stimulation does not have an effect on the latent provirus in the PHA-activated T lymphocytes (see Fig. 1) and activation of latent provirus is induced by both autologous and allogenic DCs (see Fig. 6), we chose to use antibodies that specifically target the general adhesion molecules ICAM-1, ICAM-2 or ICAM-3 (Fig. 9D). The presence of ICAM antibodies did not have an effect on the percentage of CA-p24 positive T lymphocytes in the absence of DCs (left part of the panel). The co-culture with DCs increased the percentage of CA-p24 positive T lymphocytes 5-fold. Blocking ICAM-1 interactions between the DC and T lymphocyte significantly reduced the activation by 75% to 2-fold. Addition of ICAM-2 or ICAM-3 antibodies, on the contrary, did not inhibit the DC-mediated increase in CA-p24 positive T lymphocytes. Together, these results demonstrate that both cell-cell contact between DC and T lymphocyte and (a) secreted DC factor(s) induce activation of latent HIV-1 provirus.

## Discussion

We previously developed a fast and relatively simple method to study HIV-1 latency in T cell lines and showed that the virus can be purged out of latency with TNFα, genistein and 5-Aza [28], [35]. In this study, our latency assay was used to demonstrate that HIV-1 can establish a latent proviral integration in primary PHA-activated T lymphocytes. Conventional anti-latency drugs (TNFα, NaBut, Prostratin, TSA, PMA or PHA), were not sufficient to activate the latent provirus but the virus was purged out of latency by co-culturing the T lymphocytes with dendritic cells (DCs). Co-culturing with DCs, activated gene expression from the latent HIV-1 provirus in the T lymphocytes by 2- to 5-fold. This may seem modest in comparison to other latency models [20], [29], [45], [47], [51], [52], but we note that in other studies the latently infected cells are usually selected, either by clonal selection or by specific outgrowth. In contrast, our latency assay is performed on the bulk culture without any form of selection. The obtained 2- to 5-fold activation means that for every virus-producing lymphocyte there are 1 to 4 lymphocytes that harbor a silent provirus that can be activated by DCs. In fact, since we cannot rule out that some latent proviruses are unresponsive to DC-activation, the actual latent reservoir may be underestimated.

In resting memory T lymphocytes, activation of the latent provirus is predominantly mediated via cellular activation with αCD3/CD28 (TCR-stimulation), IL-2 or via activation of the NFAT transcription factor with ionomycin [36], [45]–[47]. These stimuli did not have an effect on the silent provirus in activated T lymphocytes, most likely because the transcription factors involved are already active. This observation illustrates that the establishment of a silent provirus is not necessarily due to the absence of certain transcription factors, which was proposed to be the major cause for the establishment of latently infected quiescent memory lymphocytes [36]. Importantly, this also shows that the molecular mechanism leading to the activation of latent HIV-1 can differ between different T lymphocyte subsets, depending on their activation status.

A remaining question is whether HIV-1 latency is caused by silencing of a transcriptionally active provirus or the result of silent proviral integration. If silent integration occurs, the infected proliferating T lymphocyte will not be recognized by the immune system, favoring the transition to a latently infected memory cell. Our results showing that DCs can activate latent HIV-1 already 2 days after the initial infection support previous observations obtained in T cell lines that the latent phenotype is established immediately after provirus integration and is not due to down-regulation of an initially transcriptional active provirus [28], [29], [34].

We have not yet fully characterized the molecular interactions required for the DC-mediated HIV-1 activation from latency. However, our results indicate that both (a) secreted DC-component(s) and the cell-cell interaction between DC and T lymphocyte trigger activation of latent provirus. HIV-1 activation did not require autologous T lymphocytes and DCs, suggesting that the cell-cell interaction is not self-restricted. Blocking the DC-T cell interaction with anti-ICAM-1 antibodies strongly inhibited the activation of latent provirus. ICAM-1 is expressed on the DC and binds to leukocyte function-adhesion molecule-1 (LFA-1) on the T lymphocyte, an interaction that is important for the process of antigen presentation [53]–[55]. We and others previously showed that the interaction of LFA-1 and ICAM-1, but not ICAM-2 and ICAM-3, is crucial for efficient HIV-1 transmission and that DC subsets that express higher levels of ICAM-1 transmit HIV-1 more efficiently [56]–[58]. It would be interesting to investigate if DC subsets with higher ICAM-1 levels are also better activators of latent provirus. Further characterization of the cellular contact molecules that can purge HIV-1 from latency and testing their efficiency on latently infected memory T lymphocytes is of great interest, as our results strongly suggest that cellular activation via TCR-stimulation is not sufficient to activate all latent proviruses.

Purging the provirus from latency via physiologically relevant cell-cell interactions supports the idea that the viral reservoir is dynamic. In this study we show that immature monocyte-derived DCs can activate latent HIV-1 in activated T lymphocytes. Marini *et al.* demonstrated that mature monocyte-derived DCs can activate latent provirus in resting memory T lymphocytes [47]. If these cell-cell contacts activate latent virus *in vivo*, local outbursts of virus production can occur despite suppressive therapy. However, the situation *in vivo* is more complex as mature myeloid DCs have been reported to inhibit productive infection by inducing latency [59]. This indicates that different DC subsets may have opposing effects on viral latency. Thus, the cellular HIV-1 reservoir could be activated or silenced depending on the type of DC that is encountered. We are currently investigating the influence of different DC subsets (myeloid and plasmacytoid) on latent HIV-1 provirus in proliferating T lymphocytes.

In this study we demonstrate that HIV-1 can establish a silent integration in actively proliferating T lymphocytes. The latently infected T lymphocytes will escape immune-surveillance as long as no viral peptides are expressed and presented on the cell surface. Although proliferating T lymphocytes are generally short-lived such that their contribution to the total HIV-1 reservoir will be limited, these cells can return to the resting state of memory T lymphocyte and thereby contribute to the long-lived viral reservoir. How important this interaction is *in vivo* and whether it can be used in ‘shock and kill’ approaches [60], [61] for the complete eradication of the HIV-1 reservoir in patients remains to be studied.

## Materials and Methods

### Cells

HEK 293T cells were grown as a monolayer in Dulbecco's minimal essential medium (Gibco, BRL, Gaithersburg, MD) supplemented with 10% (v/v) fetal calf serum (FCS), 40 U/ml penicillin, 40 µg/ml streptomycin and nonessential amino acids (Gibco, BRL, Gaithersburg, MD) at 37°C and 5% CO_2_. The human T lymphocytic cell line SupT1 (ATCC CRL-1942) was cultured in advanced RPMI 1640 medium (Gibco BRL, Gaithersburg, MD) supplemented with 1% (v/v) FCS, 20 mM glucose, 40 U/ml penicillin, and 40 µg/ml streptomycin. The Raji cell line and Raji-DC-SIGN cells were cultured in RPMI 1640 medium (Gibco, BRL, Gaithersburg, MD) containing 10% FCS. The immature monocyte-derived dendritic cells (DCs) were prepared as previously described [62]. In short, human peripheral blood mononuclear cells (PBMCs) were isolated from buffy coats (Central Laboratory Blood Bank, Amsterdam, The Netherlands) by use of a Ficoll gradient. Monocytes were subsequently isolated with a CD14 selection step using a magnetic bead cell sorting system (Miltenyi Biotec GmbH, Bergisch Gladbach, Germany). Purified monocytes were cultured in RPMI 1640 medium containing 10% FCS and differentiated into DCs by stimulation with 45 ng/ml interleukin-4 (rIL-4; Biosource, Nivelles, Belgium) and 500 U/ml granulocyte macrophage colony-stimulating factor (GM-CSF; Schering-Plough, Brussels, Belgium) on day 0 and 2, and used on day 6. The remaining PBMCs were controlled by PCR for the absence of the CCR5 Δ32 allele and frozen in multiple vials. When required, the PBMCs were thawed, activated with phytohemagglutinin (PHA, Remel, 5 µg/ml for 2 days, 2 µg/ml for 3 days activation) or CD3/CD28 immunomagnetic beads (ratio cell∶beads 1∶1 for 3 days, Dynal, Invitrogen) and cultured in RPMI medium supplemented with 10% FCS and recombinant IL-2 (rIL-2, Novartis) at 100 U/ml. On day 2 of culture, CD4^+^ T lymphocytes were enriched by depleting CD8^+^ T lymphocytes using CD8 immunomagnetic beads (Dynal, Invitrogen). The CD4^+^ T lymphocytes were cultured for 3 days in RPMI medium with rIL-2 and 10% FCS.

### Virus

Plasmid DNA encoding the CXCR4-using HIV-1 LAI primary isolate [63] was transiently transfected in HEK 293 T cells with the calcium phosphate method as described previously [64]. Virus supernatant was harvested 2 days after transfection, sterilized by passage through a 0.2 µm filter and stored in aliquots at −80°C. The concentration of the virus stocks was determined by CA-p24 ELISA.

### Extracellular CA-p24 ELISA

Culture supernatant was heat inactivated at 56°C for 30 min in the presence of 0.05% Empigen-BB (Calbiochem, La Jolla, USA). The CA-p24 concentration was determined by twin-site ELISA with D7320 (Biochrom, Berlin, Germany) as capture antibody and alkaline phosphatase-conjugated anti-CA-p24 monoclonal antibody (EH12-AP) as detection antibody. Quantification was performed with the lumiphos plus system luminescence reader (Lumigen, Michigan, USA) in a LUMIstar Galaxy (BMG labtechnologies, Offenburg, Germany). Recombinant CA-p24 produced in a baculovirus system was used as standard.

### Reagents

Mannan (Sigma) was used at a final concentration of 40 µg/ml. Raltegravir (ISENSTRESS/MK-0518) was obtained through the AIDS Research and Reference Reagent Program and used at the final concentration of 50 µg/ml. The fusion inhibitor T1249 (WQEWEQKITALLEQAQIQQEKNEYELQKLDKWASLWEWF) was obtained from Pepscan (Therapeutics BV, Lelystad, The Netherlands) and used at a final concentration of 0.1 µg/ml.

### Anti-latency drugs

PHA (Remel), PMA (Sigma), Prostratin (Sigma-Aldrich), TNFα (Invitrogen), Trichostatin A (TSA, Sigma) and Sodium butyrate (NaBut, Aldrich) were used at the indicated concentrations. Recombinant IL-2 (Novartis) was used at a final concentration of 100 U/ml and ionomycin (Sigma-Aldrich) at 100 µg/ml. αCD3/CD28 beads (Dynal, Invitrogen) were used at a ratio of 1 bead per cell.

### Antibodies

For intracellular CA-p24 measurement we used the RD1- or FITC-conjugated mouse monoclonal α-CA-p24 (clone KC57, Coulter). For CD3 staining the APC- or FITC-conjugated α-CD3 (BD Bioscience) was used. Annexin V-APC (BD Pharmingen) was used to stain for the early apoptosis marker phosphatidyldserine. To characterize the T lymphocyte immunophenotye, α-CD69-PE (BD Bioscience), α-CD45-RA-PE (Pharmingen), α-CD25-FITC (BD), α-CD45-RO-FITC (DAKO), α-CD127-PE (BD Pharmingen), and α-CCR7-PE (BD Pharmingen) were used. For DC staining purified α-CD83-APC (BD Bioscience), α-CD86-PE (BD Pharmingen), α-HLA-DR-PerCPCy5 (BD Bioscience), α-CD14-FITC (BD Bioscience) and α-DC-SIGN-PE (R&D Systems) antibodies were used. To block DC-T lymphocyte cell-cell (ICAM) interactions in the co-culture, α-CD54 (Peli Cluster; the Netherlands), α-CD102 (RD Systems) or α-CD50 (Immunotech) antibodies were used.

### HIV-1 latency assay

HIV-1 infected cells were used in the latency assay as described previously [35]. In short, PHA- or CD3/CD28-activated CD4^+^ T lymphocytes (1.0 or 2.0×10^6^ cells) were infected with HIV-1 (20 ng CA-p24). Free virus was washed away after 4 hours and the cells were cultured with the fusion inhibitor T1249 to prevent new infections. At 24 hr after infection the CD4^+^ T lymphocytes (1.5×10^5^/well) were either mock treated, treated with anti-latency drugs or co-cultured with DCs, Raji or Raji-DC-SIGN cells (0.5×10^5^/well). After another 24 hr, the cells were harvested and intracellular CA-p24 was detected by FACS flow cytometry. Virus production was also determined by measuring extracellular CA-p24 in the culture supernatant by ELISA. The percentage of CA-p24 positive cells in the treated culture was divided by the percentage of CA-p24 cells in the mock treated culture and used as a measure for proviral latency (fold activation). The CD4^+^ T lymphocytes were co-cultured with autologous DCs unless when co-cultured with allogenic DC's (as indicated in the figure legend). To block cell-cell interactions between T lymphocyte and DC antibodies specific for human ICAM-1 (CD54), ICAM-2 (CD102) or ICAM-3 (CD50) were added to the co-culture at the final concentration of 10 µg/ml. To select CD4 expressing cells in the HIV-1 infected culture a magnetic bead cell sorting system was used according to the manufactures instructions (Miltenyi Biotec GmbH, Bergisch Gladbach, Germany). One Way ANOVA and student T test (2-tailed) were used to evaluate if observed differences between groups are significant (Graphpad Prism, version 5). P values * = p<0.05, ** = p<0.01, *** = p<0.001.

### FACS flow cytometry

Cells were fixed in 4% formaldehyde for 10 minutes at room temperature and subsequently washed with FACS buffer (PBS supplemented with 1% FCS). The cells were permeabilized with BD Perm/Wash buffer (BD Pharmingen) and antibody staining was performed in BD Perm/Wash or FACS buffer for 1 hr at 4°C. Excess of unbound antibody was removed and the cells were analyzed on a BD FACSCanto II flow cytometer with BD FACSDiva Software v6.1.2 (BD biosciences, San Jose, CA) in FACS buffer. The live population was defined based on forward/sideward scatter and analyzed for CD3 and intracellular CA-p24 positivity. Gate settings were fixed between samples for each experiment. To measure the membrane phospholipid phosphatidylserine as a marker for early apoptosis, the cells were stained with Annexin V prior to cell fixation.

The DC phenotype (negative for CD14, low levels of MHC class II (HLA-DR), CD83 and CD86 and high levels of DC-SIGN) was confirmed by FACS flow cytometry [57].

### Quantitative TaqMan assay

TaqMan assay was used to quantify the number of HIV-1 DNA copies in infected cultures. In summary, cells were resuspended in Tris-EDTA (10 mM pH 8.3) containing 0.5 units/µl proteinase K (Roche Applied Science), incubated for 1 hr at 56°C and 10 min at 95°C and directly used for quantitative PCR amplification. The number of input cells was determined using TaqMan reagents for quantification of β-actin DNA (AB, Applied Biosystems) according to the manufacturer's instruction. To quantitate integrated proviral DNA copy numbers a pre-amplification was done with primers detecting the repetitive Alu sequence [65] in combination with primers specific for the HIV-1 LTR. The pre-amplified DNA was subsequently quantified by real-time PCR as previously described [66].

## Supporting Information

Figure S1 **Both PHA- and CD3/CD28-activated T lymphocytes can harbor latent HIV-1 provirus.** T lymphocytes were activated via stimulation with PHA or antibodies specific for CD3 and CD28 prior to the latency assay. **A**: Co-culturing of the T lymphocytes increased the percentage of CA-p24 positive cells in both the PHA- and CD3/CD28-activated T lymphocytes. Shown is a representative graph of two independent experiments. In each experiment a different donor was used and each experiment was performed in duplicate. **B**: HIV-1 fold latency activation of the percentage of CA-p24 positive cells. The results presented are mean values (± sem) obtained from two independent experiments. In each experiment a different T lymphocyte donor was used and each experiment was performed in duplicate (n = 4).
(TIF)

Figure S2 **Immune phenotype characterization of the PHA-activated T lymphocytes.** PHA-activated T lymphocytes were infected according to the latency assay and stained with different immune phenotype markers for flow cytometry analysis. **A**: Representative mean fluorescent intensity (MFI) histogram of PHA-activated T lymphocytes expressing low levels of CD69, CD127 and CCR7 and high levels CD25 and CD45RO. **B**: Analysis of HIV-1 infected T lymphocytes; MFI expression levels of the CA-p24 negative T lymphocytes compared to the CA-p24 positive T lymphocytes. **C**: Representative MFI histogram of CA-p24 positive T lymphocytes that were co-cultured with DCs or mock treated. **D**: Analysis of the CA-p24 positive cells that were either co-cultured with DCs (+ DC) or mock treated (− DC). Results are obtained from two independently performed experiments with two different donors and each experiment was performed in triplicate (n = 6).
(TIF)

Figure S3 **CA-p24 production does not correlate with onset of apoptosis.** HIV-1 infected T lymphocytes were mock treated or co-cultured with allogenic DCs in the latency assay. Prior to fixation the cells were stained for the early apoptosis marker phosphatidylserine with Annexin-V. Subsequently the cells were fixed, stained for intracellular CA-p24 and CD3 and analyzed by flow cytometry. The forward and sideward scatter plots were used to gate the live cell population from which the CD3-positive cells were analyzed for Annexin-V and CA-p24 positivity. Shown is a representative flow cytometry figure of two independently performed experiments, in each experiment a different donor was used and each experiment was performed in triplicate (n = 6).
(TIF)

Figure S4 **HIV-1 activation from proviral latency is not induced by GM-CSF or IL-4 stimulation.** HIV-1 infected T lymphocytes were either mock treated, cultured with GM-CSF (500 U/ml), IL-4 (45 ng/ml), GM-CSF together with IL-4, or co-cultured with allogenic DCs in the latency assay. The results presented are mean values (± sem) obtained from two independent experiments. In each experiment a different T lymphocyte donor was used and each experiment was performed in triplicate (n = 6).
(TIF)
