# Supplementary figures and images for: Dendritic Cell-induced Activation of Latent HIV-1 Provirus in Actively Proliferating Primary T Lymphocytes
Source: PLoS Pathog. 2013 Mar 21;9(3):e1003259. doi: 10.1371/journal.ppat.1003259 (PMC3605277; doi:10.1371/journal.ppat.1003259)

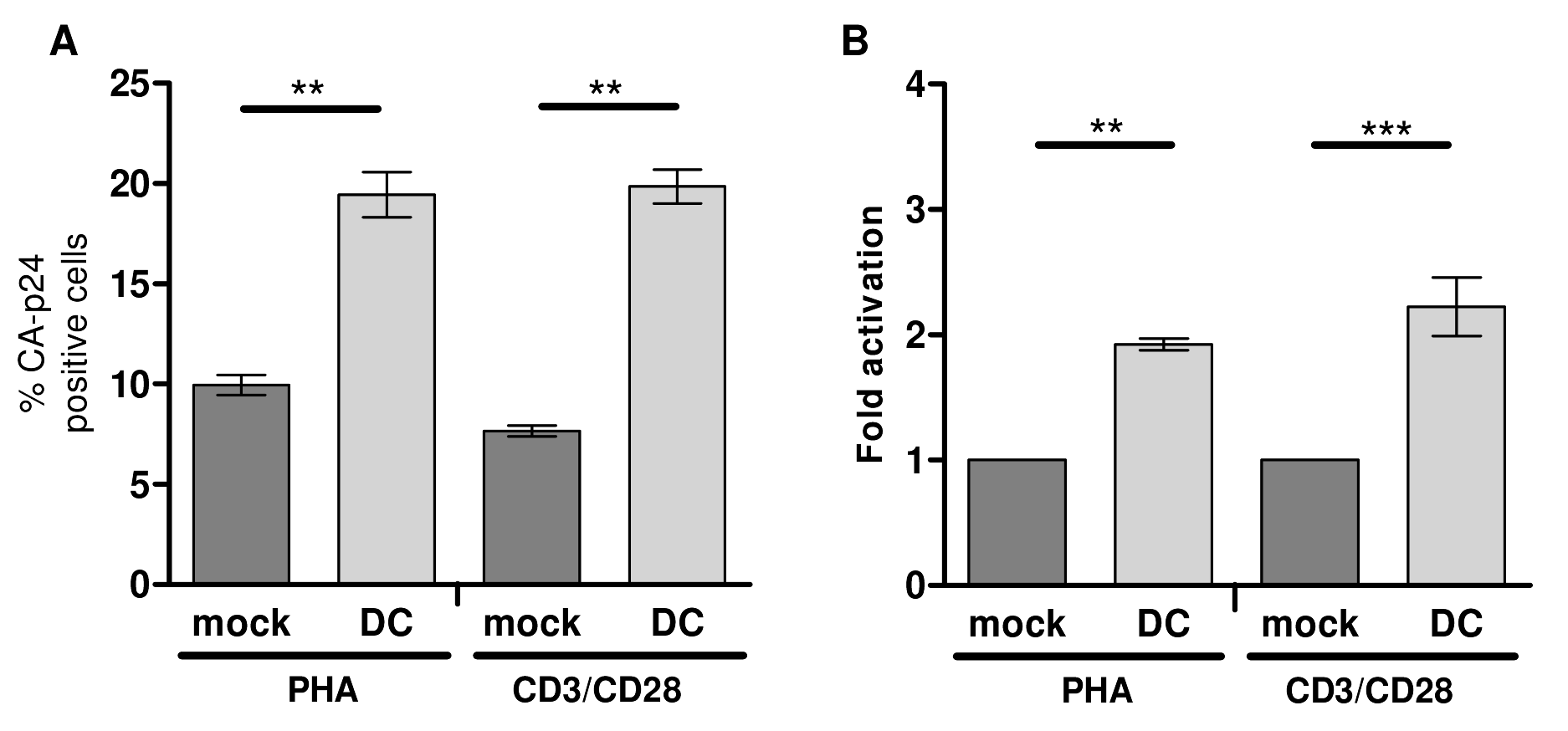

Supplement: Figure S1 — Both PHA- and CD3/CD28-activated T lymphocytes can harbor latent HIV-1 provirus. T lymphocytes were activated via stimulation with PHA or antibodies specific for CD3 and CD28 prior to the latency assay. A: Co-culturing of the T lymphocytes increased the percentage of CA-p24 positive cells in both the PHA- and CD3/CD28-activated T lymphocytes. Shown is a representative graph of two independent experiments. In each experiment a different donor was used and each experiment was performed in duplicate. B: HIV-1 fold latency activation of the percentage of CA-p24 positive cells. The results presented are mean values (± sem) obtained from two independent experiments. In each experiment a different T lymphocyte donor was used and each experiment was performed in duplicate (n = 4). (TIF) [file ppat.1003259.s001.tif]

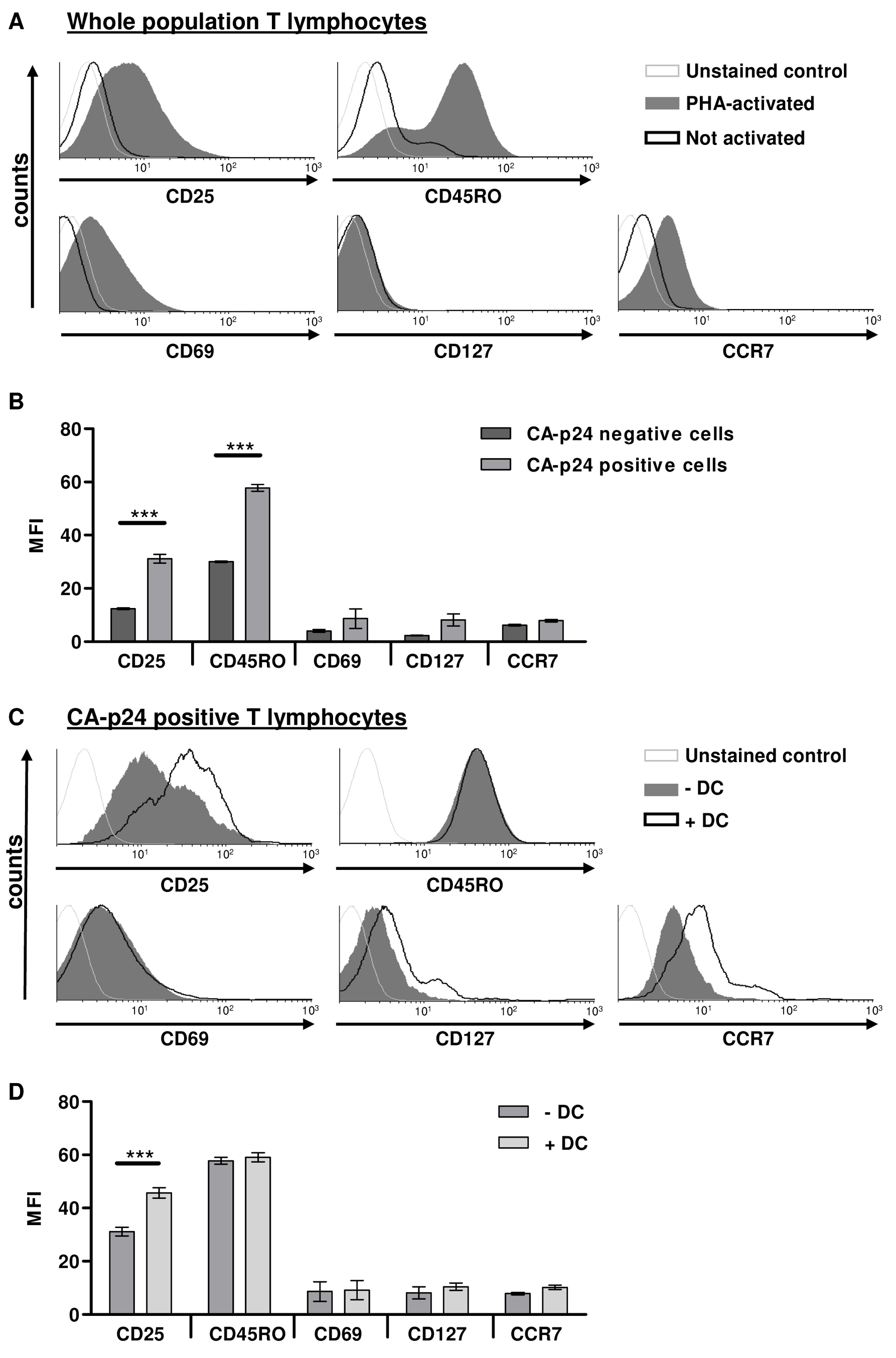

Supplement: Figure S2 — Immune phenotype characterization of the PHA-activated T lymphocytes. PHA-activated T lymphocytes were infected according to the latency assay and stained with different immune phenotype markers for flow cytometry analysis. A: Representative mean fluorescent intensity (MFI) histogram of PHA-activated T lymphocytes expressing low levels of CD69, CD127 and CCR7 and high levels CD25 and CD45RO. B: Analysis of HIV-1 infected T lymphocytes; MFI expression levels of the CA-p24 negative T lymphocytes compared to the CA-p24 positive T lymphocytes. C: Representative MFI histogram of CA-p24 positive T lymphocytes that were co-cultured with DCs or mock treated. D: Analysis of the CA-p24 positive cells that were either co-cultured with DCs (+ DC) or mock treated (− DC). Results are obtained from two independently performed experiments with two different donors and each experiment was performed in triplicate (n = 6). (TIF) [file ppat.1003259.s002.tif]

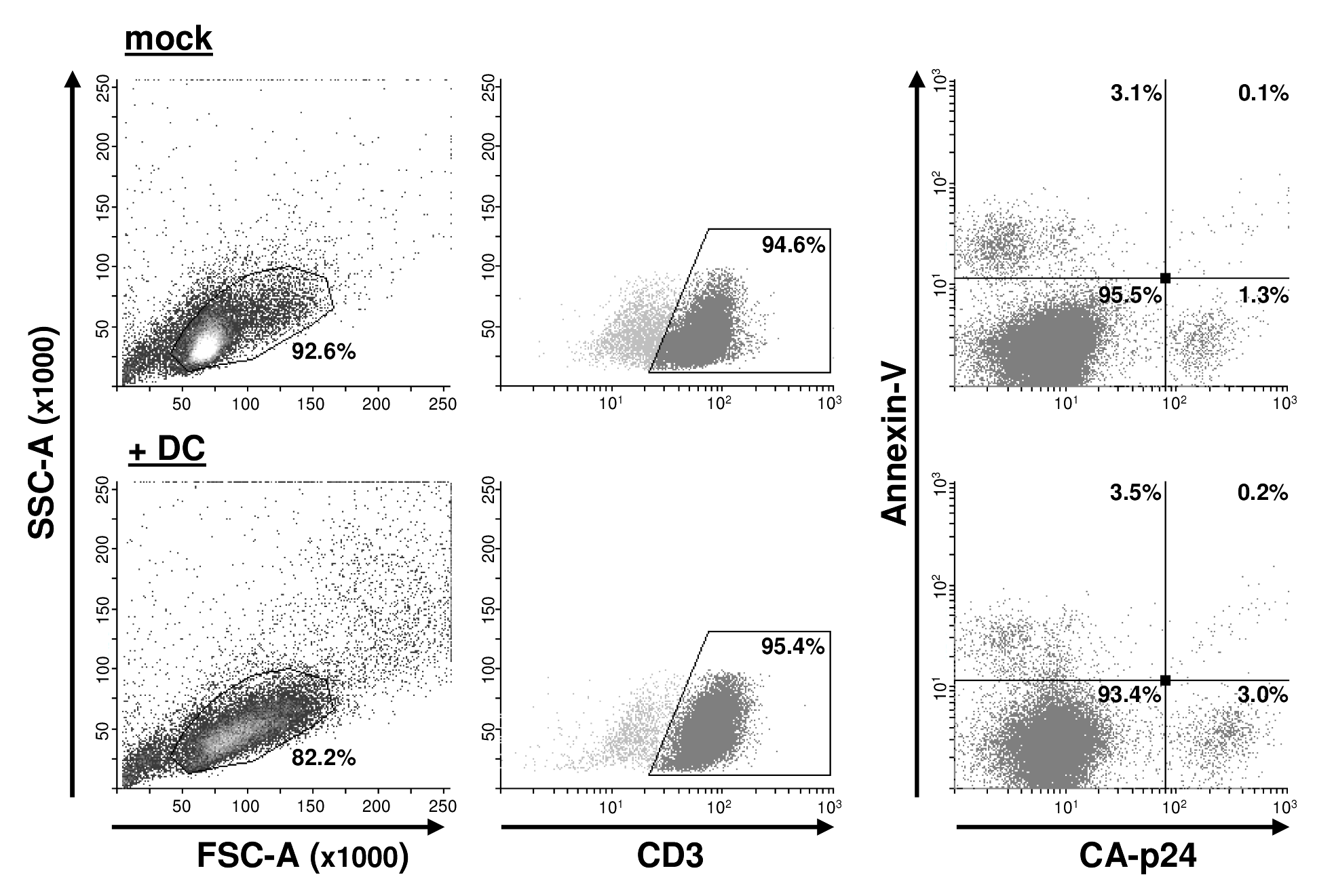

Supplement: Figure S3 — CA-p24 production does not correlate with onset of apoptosis. HIV-1 infected T lymphocytes were mock treated or co-cultured with allogenic DCs in the latency assay. Prior to fixation the cells were stained for the early apoptosis marker phosphatidylserine with Annexin-V. Subsequently the cells were fixed, stained for intracellular CA-p24 and CD3 and analyzed by flow cytometry. The forward and sideward scatter plots were used to gate the live cell population from which the CD3-positive cells were analyzed for Annexin-V and CA-p24 positivity. Shown is a representative flow cytometry figure of two independently performed experiments, in each experiment a different donor was used and each experiment was performed in triplicate (n = 6). (TIF) [file ppat.1003259.s003.tif]

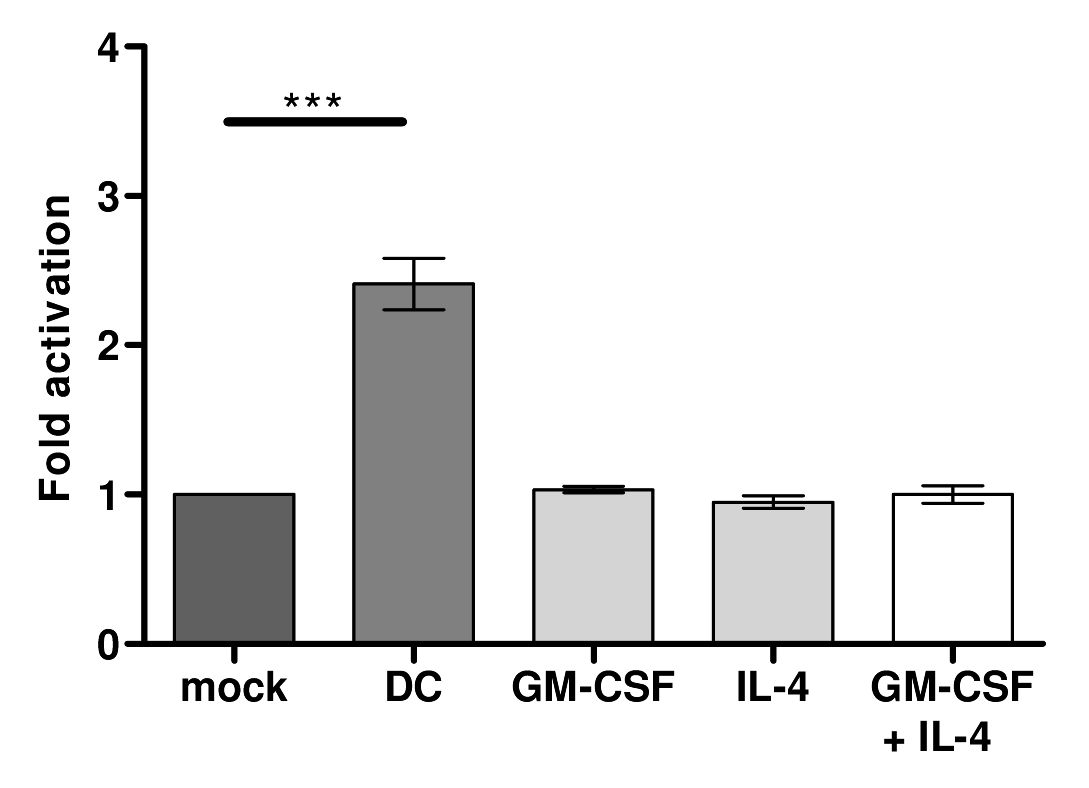

Supplement: Figure S4 — HIV-1 activation from proviral latency is not induced by GM-CSF or IL-4 stimulation. HIV-1 infected T lymphocytes were either mock treated, cultured with GM-CSF (500 U/ml), IL-4 (45 ng/ml), GM-CSF together with IL-4, or co-cultured with allogenic DCs in the latency assay. The results presented are mean values (± sem) obtained from two independent experiments. In each experiment a different T lymphocyte donor was used and each experiment was performed in triplicate (n = 6). (TIF) [file ppat.1003259.s004.tif]
